# Hypoxia Acclimation Protects against Heart Failure Postacute Myocardial Infarction via Fundc1-Mediated Mitophagy

**DOI:** 10.1155/2022/8192552

**Published:** 2022-04-05

**Authors:** Qin Li, Yinghai Liu, Qingqing Huang, Xiaobo Yi, Fuen Qin, Zuling Zhong, Lu Lin, Haihong Yang, Gu Gong, Wei Wu

**Affiliations:** Department of Anesthesiology and Pain Medicine, The General Hospital of Western Theater Command, Sichuan, Chengdu, China

## Abstract

Mitochondrial dysfunction is the main cause of heart failure (HF) postacute myocardial infarction (AMI). Hypoxia acclimation (HA) reduces efficiently the area of AMI caused by ischemia and/or reperfusion and delays HF. Here, we examined whether HA improves mitochondrial structure and function through the hypoxic autophagy receptor FUNDC1 to prevent HF post-AMI. Male adult mice were acclimated in a low-pressure hypoxic animal chamber (11% oxygen (O_2_)) for 8 h/day for 28 days, and then, an induced HF post-AMI model via left anterior descending (LAD) artery ligation was structured to explore the efficacy and mechanism of HA. Our results showed that HA exposure can improve cardiac structure and function in mice with HF post-AMI and protected myocardial mitochondrial morphology and function. Further studies showed that HA increased the expression of Fundc1 protein and its associated mitophagy protein LC3 in myocardial tissue after infarction. We then established a cellular model of oxygen glucose deprivation (OGD) in vitro, and knockdown of FUNDC1 attenuated the protective effect of HA exposed on cardiomyocyte mitochondria and increased cardiomyocyte apoptosis. In conclusion, the protective effect of HA on HF post-AMI is achieved by regulating Fundc1-mediated mitophagy in myocardial tissue. FUNDC1-mediated mitophagy could be a promising strategy to treat cardiovascular diseases, including HF.

## 1. Introduction

With recent advances in cardiovascular drugs and interventional therapy for AMI, the survival of patients' HF post-AMI has improved [1]. However, the damaged, terminally differentiated cardiomyocytes have limited ability to regenerate. HF post-AMI remains a major cause of cardiovascular disease morbidity and mortality in humans [2, 3]. Mitochondria, as signaling hubs, are important targets in the complex pathogenic factors of cardiac ischemic injury and heart failure. Under physiological conditions, mitochondria produce 90% of ATP in the heart and are involved in regulating glucose and fat metabolism, maintaining cellular ion homeostasis, and laying the foundation for normal cell function [4, 5]. However, when ischemic damage happened in cardiomyocytes, mitochondrial damage bears the brunt. The main features of mitochondrial damage include ATP deficiency, alterations of substrate utilization, dysfunction of OXPHOS, excessive accumulation of reactive oxygen species, impaired inflammation, and metabolic signals [6–8]. Excessive mitochondrial damage triggers cell death pathways, often culminating in tissue collapse [9]. To prevent such excessive mitochondrial damage and ensure cardiac mitochondrial homeostasis and function, the dysfunctional mitochondria are eliminated by mitophagy [10].

Autophagy is a lysosomal-mediated catabolic pathway that is responsible for eliminating misfolded proteins and dysfunctional organelles to promote cell survival and maintain intracellular energy homeostasis [11]. Autophagy can be employed by cells to degrade cellular contents nonspecifically or to specifically target dysfunctional organelles. Mitophagy is the process of selectively targeting mitochondria for autophagy, and it is essential for clearing defective or damaged mitochondria [12]. Thus, optimization of mitochondrial function and structure through mitophagy can improve cardiac function, rescue damaged cardiomyocytes, and provide protection against ischemic heart disease. Mitophagy is regulated via receptor-mediated FUN14 domain-containing protein 1 (Fundc1) or Bnip3 or receptor-independent PINK1/Parkin pathways [13–15]. Among them, FUNDC1 is a newly discovered important molecule for receptor-dependent mitophagy in recent years. It is an outer mitochondrial membrane protein with a characteristic structure of LIR motif (Y18-EV-L21). The induced mitophagy does not require labeling. Damaged mitochondria can directly interact with LC3 on the phage through LIR to identify damaged mitochondria and transfer them to autophagic vesicles, thus triggering mitophagy [16, 17]. Current studies have shown that hypoxia-activated Fundc1-mediated mitophagy is essential for restoring mitochondrial function and cell/organelle balance in myocardial ischemia/reperfusion injury [18, 19]; moreover, emerging evidence suggests that autophagy is involved in cardioprotection [10].

Epidemiological studies have demonstrated that people living at high altitudes are less likely to suffer from chronic ischemic heart disease and present a lower heart disease-related death rate [20]. Therefore, the simulation of high altitude conditions, especially hypoxic (low oxygen) and hypobaric (low ambient air pressure) conditions, has been the focus of the latest research on post-MI functional recovery [21]. Several studies have demonstrated that intermittent hypoxia exposure (equivalent to an altitude of 5,000 m, for 6-8 h period each day for 28 to 42 days) has been widely accepted as a powerful and reproducible remedy for cardioprotection [22–24]. *In vivo* and *in vitro* experiments have shown that HA-exposed cardiac protection may involve multiple mechanisms, such as increasing myocardial capillary density and coronary blood flow, inhibiting mitochondrial pore opening, maintaining calcium channels, enhancing the ability to resist calcium overload, and inhibiting endoplasmic reticulum stress [23, 25]. In addition, HA-exposed hearts can alleviate cardiac I/R injury through elevated protein O-GlcNAcylation, enhanced pentose phosphate pathway, and improved redox homeostasis [26]. Recently, exposure to stimuli, such as inhaled anesthetics, hypoxic conditions, and transient ischemia, was found to induce autophagy and possibly also confer cardioprotection against ischemia-reperfusion injury in animal experiments [27, 28]. At present, little is known about changes in Fundc1 levels and Fundc1-mediated mitophagy in ischemic myocardial tissue under HA.

Therefore, considering the abovementioned researches and the fact that Fundc1 is involved in the response to hypoxia and in autophagy, we established an animal model of HA to explore hypoxic conditions inducing mitophagy and contributing to the removal of damaged mitochondria, thus maintaining myocardial cellular homeostasis and function, and to identify potential targets for improved MI therapies.

## 2. Materials and Methods

### 2.1. Mice

**Figure 1:**
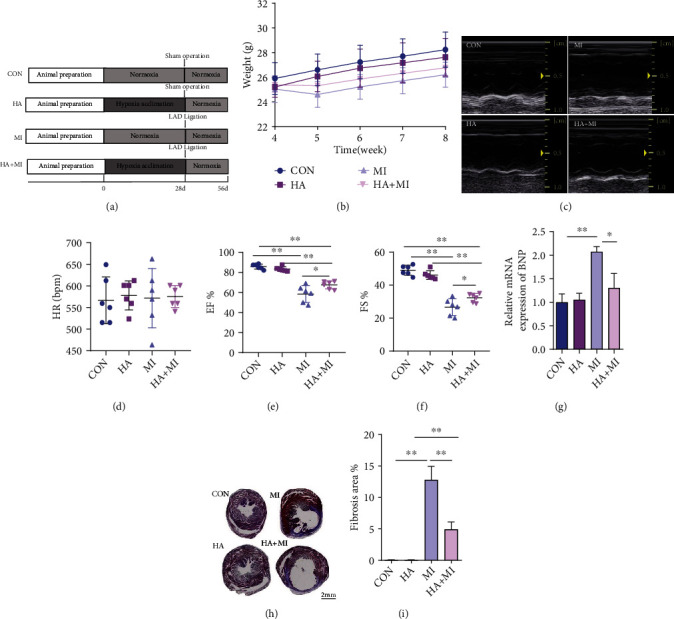
HA improves cardiac function and structure after MI injury. (a) Schematic showing the experimental protocol and workflow. (b) The trend of body weight changes after MI (*n* = 8-10 per group). (c) Representative images of M-mode echocardiography after MI (*n* = 6 per group). (d) Mouse heart rate (HR) (beats per minute (bpm)) (*n* = 6 per group). (e, f) Mouse ejection fraction percentage (EF%) and fractional shortening percentage (FS%) (*n* = 6 per group). (g) Relative BNP mRNA expression in the CON, HA, MI, and HA+MI groups after MI, as determined by qPCR (*n* = 3 per group). (h) Cardiac fibrosis detected by Masson's trichrome staining of paraffin-embedded cardiac tissue sections. Representative images are shown. Scale bars, 2 mm. (i) Quantification of the fibrosis area relative to the left ventricle (*n* = 3 per group). All data were expressed as mean ± SD. ^∗^*P* < 0.05, ^∗∗^*P* < 0.01 vs. control (CON) group.

All animal experiments were approved by the experimental Animal Care and Use Committee of the General Hospital of Western Theater Command and complied with *the Guide for the Care and Use of Laboratory Animals* published by the US National Institutes of Health (NIH publication, 8th edition, 2011). Male C57BL/6 mice (8–10 weeks old), weighing 20 ± 5 g, were purchased from the Chengdu Dashuo Experimental Animal Center (Sichuan, China). Mice were maintained in a vivarium with a 12 h light/dark cycle at 22°C and free access to food and water. Mice were randomly assigned to be subjected to normal hypoxia and sham surgery (CON), hypoxia acclimation and sham surgery (HA), normal hypoxia and AMI surgery (MI), or hypoxia acclimation and MI surgery (HA+MI). The experimental workflow and each experimental group are illustrated in Figure 1(a).

### 2.2. Cell Culture

The H9C2 rat cardiomyocyte cell line was obtained from the American Type Culture Collection (ATCC, Rockville, MD, USA). The cells were cultured in low-glucose DMEM (HyClone Laboratories, Inc., Chengdu, Sichuan, China) supplemented with 10% (*v*/*v*) fetal bovine serum (Biological Industries), 100 U/mL penicillin, and 100 mg/mL streptomycin (both from HyClone Laboratories, Inc., Chengdu, Sichuan, China). To modulate Fundc1 expression level, the culture medium was replaced with transfection medium containing the Fundc1 shRNA-bearing adenovirus (Cyagen Biosciences Inc., Chengdu, Sichuan, China; multiplicity of infection (MOI) = 200), and the cells were transfected in the incubator for 24 h at 37°C, 5% CO_2_. Fundc1 knockdown efficiency was verified by qPCR.

### 2.3. Hypoxia Acclimation Exposure

To perform HA, a low-pressure hypoxic animal chamber (FLYDWC50, Feng Lei, Guizhou, China) was used to simulate 5,000 m altitude (PB = 404 mmHg, PO_2_ = 84 mmHg, 8 h/day (from 9:00 a.m. to 17:00 p.m.), 28 days) [29]. For the cell culture study, cardiomyocytes were placed into a hypoxia incubator (11% O_2_, 5% CO_2_, and 84% N_2_) for 8 h/day and then switched to normoxia (20% O_2_, 5% CO_2_, and 75% N_2_) for 16 h/day, and this treatment was repeated for 3 days.

### 2.4. Mouse Model of Myocardial Infarction Injury and Cell OGD Model

To create a mouse heart failure model, permanent left anterior descending coronary artery occlusion was used to induce myocardial infarction [30]. Mice were anesthetized by intraperitoneal injection of 2,2,2-tribromoethanol (0.5 mg/g) and then intubated with an 18-gauge intra-arterial catheter. A rodent ventilator (Model 845, Harvard Apparatus) with 60% oxygen was used during the surgical procedure. Following anesthesia, the chest was opened by a horizontal incision via the space between the fourth and fifth ribs, and the LAD coronary artery was ligated with a 6-0 silk suture. Successful ischemia was verified by the presence of a pale myocardium and dyskinesis of the ischemic region. The sham operation group performed the same operation, but the LAD coronary artery was not ligated. For cell culture studies, the OGD model was established by placing H9C2 cells in low-glucose DMEM (Gibco, Inc., Chengdu, Sichuan, China) and hypoxic environment (1% O_2_, 5% CO_2_, and 94% N_2_) for 6 hours. [31].

### 2.5. *In Vivo* Echocardiographic Measurements

We assessed mouse cardiac function at 28 days after HA or MI using a small-animal echocardiographic imaging system (Vivi7 Dimension, GE, Boston, USA). Mice were anesthetized with 1.5% isoflurane, and two-dimensional echocardiographic views of the midventricular short axis were obtained at the level of the papillary muscle tips, below the mitral valve. Left ventricular (LV) wall thickness and internal dimensions were measured, and LV fractional shortening (FS) and ejection fraction (EF) were calculated as previously described [32]. During the echocardiography, all mouse heart rates were maintained in the range of 500 beats/min. Echocardiographic imaging and measurements were performed by a professional technician who was blinded to the experimental groups.

### 2.6. Histopathology

Following the HA or MI procedures, mice were humanely euthanized at 28 days post-MI, and cardiac tissue samples were collected. The tissue samples were fixed with 10% neutral buffered formalin, paraffin embedded, and cut into 5 *μ*m thick sections. Sections were prepared for hematoxylin and eosin (H&E) staining to measure the extent of myocardial necrosis, interstitial edema, and cellular swelling. In addition, sections were stained using Masson's Trichrome Stain Kit (Solarbio, Beijing, China), according to the manufacturer's instructions, to evaluate the infarct size. The stained slides were observed with an optical microscope (Zeiss, Oberkochen, Germany). The images were digitized using the Image-Pro Plus software (Media Cybernetics, USA) and analyzed for myocardial fibrosis.

### 2.7. Transmission Electron Microscopy

The mitochondrial ultrastructure in the myocardial tissue adjacent to the MI was analyzed by transmission electron microscopy. The cardiac tissue was fixed using 3% glutaraldehyde in 0.1 M phosphate buffer saline (pH 7.4) for 24 h, and then fixed with 1% osmium tetroxide for 1 h. After the samples were dehydrated in a series of ethanol and were embedded in Epon 812, ultrathin sections were prepared and counterstained with uranyl acetate and lead citrate. The samples were visualized using a transmission electron microscope (HT7700, HITACHI, Japan), and images were captured in a blinded fashion by pathologists.

### 2.8. Flow Cytometry

Cell survival and apoptosis were assessed by flow cytometry with Annexin V-fluorescein isothiocyanate and propidium iodide (Annexin V-FITC Apoptosis Detection Kit, Shanghai, China). Briefly, H9C2 cells were harvested by treatment with ethylenediaminetetraacetic acid-free trypsin, followed by three washes with PBS. The harvested cells were mixed with 5 *μ*L Annexin V-fluorescein isothiocyanate and 5 *μ*L propidium iodide and incubated at room temperature for 5 min in the dark. The apoptosis rate was determined by flow cytometry analysis (Annexin V-FITC, Ex/Em: 488 nm/525 nm; PI, Ex/Em: 561 nm/575 nm).

### 2.9. Lactate Dehydrogenase (LDH) Release

LDH is a stable enzyme that is released from the cell cytosol into the culture medium as a consequence of cellular damage. Thus, we analyzed the amount of LDH released to the culture medium using an automatic biochemical analyzer (Miri Medical International Ltd., Shenzhen, Guangdong, China), as an indirect measurement of cell injury or damage.

### 2.10. Measurement of Mitochondrial Oxygen Consumption Rates (OCR)

The OCRs of isolated intact mitochondria or H9C2 cells were measured with a Seahorse XFe24 analyzer (Agilent Technologies, Santa Clara, CA). Mitochondria from cardiac tissue were isolated by differential centrifugation as described previously [33]. Freshly isolated cardiac mitochondria were transferred to a XFe24 microplate, and the microplate was filled with Mitochondrial Assay Solution (MAS: 220 mM D-mannitol, 70 mM sucrose, 10 mM MgCl_2_, 2 mM HEPES, 2 mM EGTA, 10 mM KH_2_PO_4_, and BSA 0.025% (*w*/*v*), pH 7.2 at 37°C) to a final amount of 500 *μ*L containing 10 mM glutamate and 2 mM malate. The OCR was measured in response to sequential injection of 4 mM adenosine 5′-diphosphate sodium salt (ADP; Sigma), 2.5 *μ*M oligomycin (ATP synthase inhibitor; MCE), 4 *μ*M carbonyl cyanide 4-(trifluoromethoxy) phenylhydrazone (FCCP, mitochondrial respiration uncoupler (MCE)), and 4 *μ*M antimycin A (complex III inhibitor; Sigma), at 37°C. Basal respiration before ADP injection represented the state 2 respiration, and maximum respiration after ADP injection represented state 3 respiration. The respiratory control ratio (RCR) was calculated by dividing state 3 respiration by state 2 respiration [34]. Maximal respiration was equal to the maximum rate measured after FCCP injection. All readings are normalized to *μ*g of mitochondrial protein.

For the measurement of cellular OCR, cardiomyocytes (2 × 10^5^ cells/well) were seeded in 24-well XFe24 cell culture microplates and treated as described above. Basal OCR was determined in cardiomyocytes cultured in the XF base medium, which consists of modified DMEM (pH 7.4) supplemented with 1 mM pyruvate, 2 mM L-glutamine, and 10 mM glucose, and incubated in a CO_2_-free environment for 1 h. OCR was also quantified after adding 10 mM oligomycin, 30 *μ*M FCCP, and 10 mM rotenone (complex I inhibitor)/10 mM antimycin A through ports in the Seahorse Flux Park cartridges. OCR was determined by the XF Cell Mito Stress Test Generator by Seahorse Bioscience. The OCR values were standardized according to the cell number.

### 2.11. Western Blot Analysis

The LV tissue and cardiomyocytes were washed with cold PBS and lysed with RIPA buffer containing a protease inhibitor cocktail. Lysates were centrifuged at 14,000 × *g* for 15 min at 4°C. Protein concentration was quantified using the BCA Protein Assay Kit (Thermo Fisher Scientific, Waltham, MA). The proteins in the lysate (10–60 *μ*g) were separated by electrophoresis, and the bands were transferred to PVDF membranes. The membranes were incubated for 2 h in 5% nonfat dry milk to block nonspecific antibody binding to the membrane and then overnight at 4°C with primary antibodies. The primary antibodies used were as follows: anti-Fundc1 (1 : 1,000, Abcam, #ab224722), anti-LC3B (1 : 1,000, Cell Signaling Technology, #2775S), anti-Pink1 (1 : 1,000, Abcam, #ab23707), and anti-Parkin (1 : 1,000, Proteintech, #23272-1-AP). The membranes were washed with Tris-buffered saline solution containing 0.1% Tween 20 (TBST, pH 7.6) and then probed with appropriate secondary antibodies (1 : 1,0000, Cell Signaling Technology, #5151S or #5257S) at room temperature for 90 min. For use as internal controls, GAPDH and *β*-actin levels were quantified using specific antibodies (anti-GAPDH, 1 : 1,0000, Proteintech, #10494-1-AP; anti-*β*-actin, 1 : 1,000, Cell Signaling Technology, #4970S). The protein bands were detected using a Bio-Rad imaging system (Hercules, CA, USA).

### 2.12. RNA Isolation and Quantitative Real-Time PCR (qPCR)

Total RNA was extracted from frozen heart tissue or cultured cells using the TRIzol reagent (Invitrogen, USA) and reverse-transcribed into complementary DNA (cDNA) using the iScript™ cDNA Synthesis kit (Bio-Rad Laboratories, Inc.). All qPCR reactions were performed using 2 *μ*L cDNA, 5 *μ*L SYBR Green Master Mix (Bio-Rad Laboratories, Inc.), and 1 *μ*L primer mix in a total volume of 10 *μ*L. Transcript levels were measured using the Bio-Rad CFX real-time quantitative PCR system (two-channel real-time PCR instrument, Bio-Rad, USA). The qPCR primers were as follows:

GAPDH: forward 5′-ATGGGAAGCTGGTCATCAAC-3′ and reverse 5′-GTGGTTCACACCCATCACAA-3′

HIF-1*α*: forward 5′-CATAAAGTCTGCAACATGGAAGGT-3′ and reverse 5′-ATTTGATGGGTGAGGAATGGGTT-3′

BNP: forward 5′-ACAATCCACGATGCAGAAGCT-3′ and reverse 5′-GGGCCTTGGTCCTTTGAGA-3′

FUNDC1: forward 5′-CCCCTCCCCAAGACTATGAAAG-3′ and reverse 5′-AGGAAACCACCACCTACTGC-3′

### 2.13. Statistical Analysis

Data were analyzed by using GraphPad Prism 5.0 (GraphPad, La Jolla, CA), and the Shapiro-Wilk normality test was performed to determine data distribution. All data are normally distributed and presented as mean ± SD. Differences between two groups were analyzed using the unpaired Student's two-tailed *t-*test. Comparisons between more than two groups were evaluated using one-way ANOVA. Multiple comparisons were performed between groups using Tukey's multiple comparison test. Results with *P* < 0.05 were considered statistically significant.

## 3. Results

### 3.1. The Effects of HA on Mouse Body Weight, Heart Structure, and Ventricular Function

**Figure 2:**
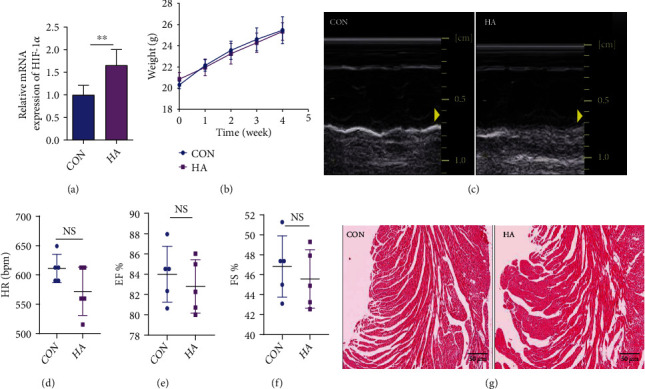
Hypoxia acclimation (HA) pretreatment has no effect on mouse heart function and structure compared with that in the control (CON) group. (a) Hypoxia-inducible factor- (HIF-) 1*α* expression in mouse heart tissues from the HA and CON groups, as determined by qPCR (*n* = 4-5 per group). (b) Effect of HA on body weight (*n* = 20 per group). (c) Cardiac function assessment by echocardiography after HA pretreatment (*n* = 5 per group). (d) Mouse heart rate (HR) (beats per minute (bpm)) (*n* = 5 per group). (e, f) Mouse ejection fraction percentage (EF%) and fractional shortening percentage (FS%) (*n* = 5 per group). (g) Hematoxylin and eosin staining of mouse left ventricular (LV) tissues from HA and CON groups. Scale bar, 50 *μ*m. All data are expressed as mean ± SD. ^∗^*P* < 0.05, ^∗∗^*P* < 0.01 vs. control (CON) group.

HA treatment significantly increased the expression of the classic hypoxia-inducible factor HIF-1*α* compared with the CON group (Figure 2(a)), indicating the establishment of hypoxic conditions, while HA did not affect mouse growth body weight (Figure 2(b)). In the absence of differences in mouse heart rates, echocardiograms of the mouse heart indicated that the left ventricular EF percentage (EF%) and FS percentage (FS%) of the HA group measured were not different from those of the CON group (Figures 2(c)–2(f)). In the H&E-stained LV sections, signs of myocardial histopathological damage, such as loss of the nucleus, cell swelling, and vacuole formation, were not considerable in the HA group (Figure 2(g)).

### 3.2. The Effects of HA on Mouse Body Weight, Ventricular Function, and Cardiac Infarct Size after MI

In order to further observe whether HA pretreatment can prevent HF-related cardiac dysfunction after MI injury, we established a MI mouse model. We noticed that the mice lost weight in the first week after MI compared to the mice in the sham group. Simultaneously, the weight loss of mice in HA+MI was lower than that in MI. Over the next three weeks, mice in the surgery group tended to gain weight slower than those in the sham group (Figure 1(b)). Similarly, the EF% and FS% were decreased after MI by echocardiography, suggesting that the left ventricular function was impaired by MI. However, the recovery of EF% and FS% after MI was improved in HA mice compared with the MI group (Figures 1(c)–1(f)). Furthermore, the HA+MI group exhibited significantly diminished B-type natriuretic peptide (BNP) expression, an indicator of cardiac damage, in the infarcted myocardium (Figure 1(g)). The myocardial infarct size of HA+MI mice was also decreased compared with that of MI mice by Masson's trichrome staining (Figures 1(h) and 1(i)).

### 3.3. The Effects of HA on Mitochondrial Function and Structure of the Heart after MI

**Figure 3:**
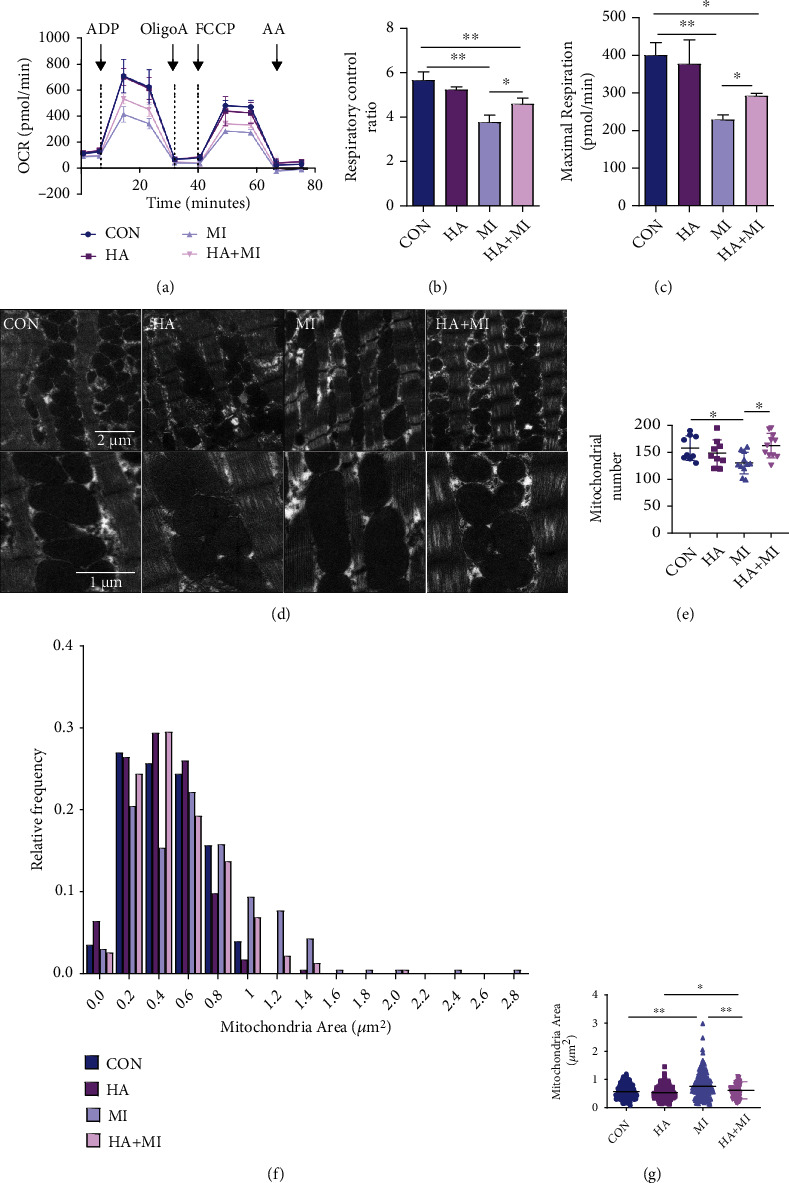
The effects of HA pretreatment on mitochondrial function and structure of heart after MI. (a–c) Myocardial tissue mitochondrial respiratory control ratio (RCR) and maximal respiratory rate were examined in post-MI and sham-treated mice exposed to normoxia or HA (*n* = 3 per group). (d) Transmission electron microscopic images showing mitochondrial structure of myocardial cells at the junction of the left ventricular ligation site after MI. (e–g) Number (scale: 5 *μ*m) and area (scale: 1 *μ*m) of mitochondria in each group. Data are expressed as mean ± SD. ^∗^*P* < 0.05, ^∗∗^*P* < 0.01 vs. control (CON) group.

To further determine whether the HA pretreatment had protective effect on mitochondria with MI injury-induced dysfunction, mitochondrial oxygen consumption rates involved in cardiac mitochondrial respiration were determined using seahorse. After MI, glutamate-induced mitochondrial RCR was lower in the MI and HA+MI mice than in the CON mice. However, the glutamate-induced mitochondrial RCR after MI was improved in HA mice compared with the MI mice (Figures 3(a) and 3(b)). Similarly, maximal mitochondrial respiration was reduced in the MI group and HA+MI group compared with the CON group, and the HA+MI group partially eliminated this inhibitory effect compared with the MI group (Figures 3(a) and 3(c)). Subsequently, the mitochondrial structure was observed by using a transmission electron microscope (Figure 3(d)). It was shown that the number and area of mitochondria in the MI group were lower than those in the CON group and higher. In contrast, compared with the HA+MI group, the number and area of mitochondria in the MI group were lower and higher, respectively (Figures 3(e)–3(g)).

### 3.4. The Effects of HA on Mitophagy Induced by Fundc1

**Figure 4:**
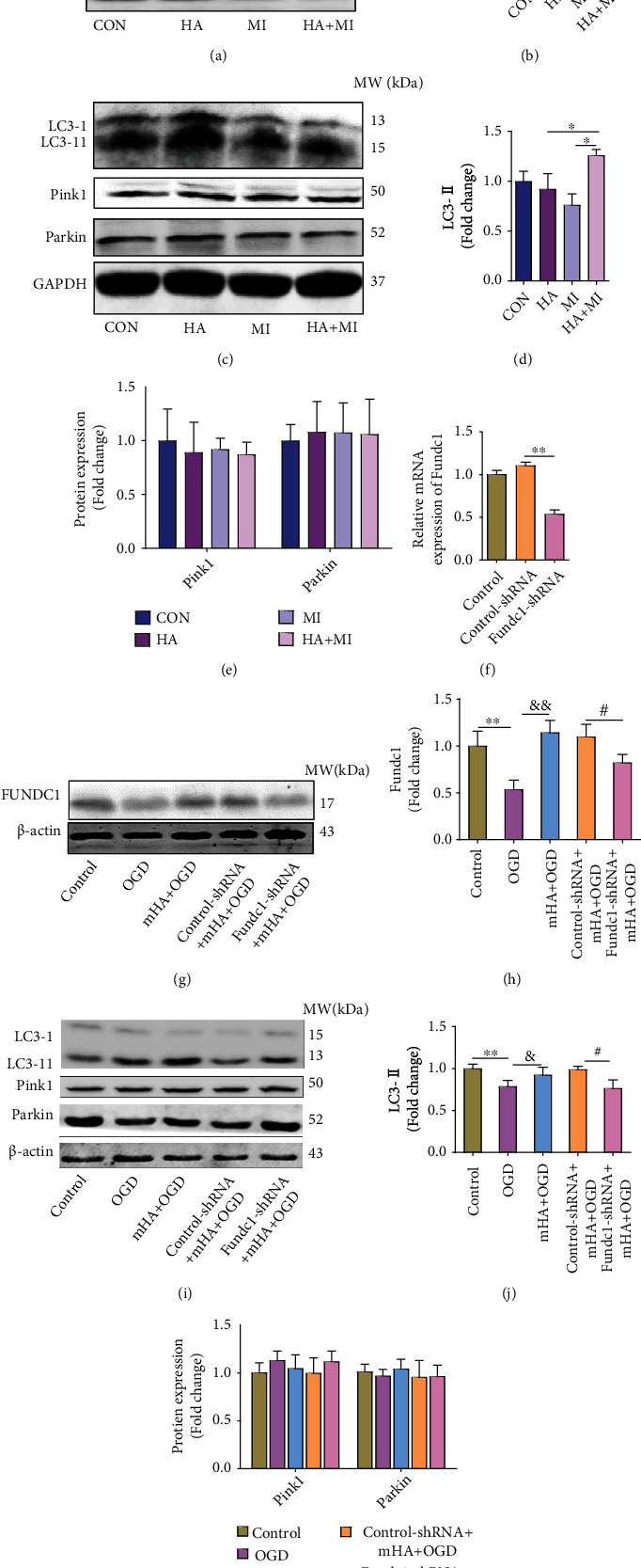
HA pretreatment activated Fundc1-mediated mitophagy after MI. (a) Fundc1 expression in the post-MI and the sham groups exposed to normoxia or HA, as determined by western blot analysis. (b) Relative Fundc1 protein expression in the HA, MI, and HA+MI groups, as determined by optical density analysis (*n* = 5-6 per group). (c) Expression of LC3, Pink1, and Parkin in the MI- (post-MI) and the sham-treated mice exposed to normoxia or HA, as determined by western blot. (d, e) Relative LC3, Pink1, and Parkin protein expression in the HA, MI, and HA+MI groups, as determined by optical density analysis (*n* = 3-5 per group). (f) H9C2 cells were transfected with Fundc1 or control-shRNA, and Fundc1 mRNA levels were measured by qPCR (*n* = 3 per group). (g) Western blot analysis of Fundc1 protein expression in cardiomyocytes from each group. (h) Relative Fundc1 protein expression in the groups analyzed in (g), as determined by optical density analysis (*n* = 5 per group). (i) Western blot analysis of LC3, Pink1, and Parkin protein levels. (j, k) Relative expression of the proteins analyzed in (i), as determined by densitometry (*n* = 3-6 per group). Data are expressed as mean ± SD (*n* = 3–5). ^∗^*P* < 0.05, ^∗∗^*P* < 0.01 vs. control, ^&^*P* < 0.05, ^&&^*P* < 0.01 vs. OGD, ^#^*P* < 0.05, ^##^*P* < 0.01 vs. control-shRNA+mHA+OGD. mHA: mimicked hypoxia acclimation; OGD: oxygen glucose deprivation.

To further observe whether the HA pretreatment changes in Fundc1 expression and Fundc1-mediated mitophagy could reverse HF-related mitochondrial dysfunction after MI injury. Protein expression involved in mitophagy was determined using western blot. As shown in Figures 4(a) and 4(b), the Fundc1 protein level of the MI group was lower than that of the CON group. However, the expression of Fundc1 in HA+MI increased compared with that in the HA group or CON group. At the same time, compared with the MI group, the expression of Fundc1 in the HA+MI group was significantly increased (Figures 4(a) and 4(b)). We next determined the expression of the active lipidated form of LC3-II, which is a known marker of autophagy. The expression of LC3-II was the highest in the HA+MI group, followed by the HA and MI groups (Figures 4(c) and 4(d)). In addition, the levels of other proteins related to mitophagy, such as Pink1 and Parkin, were not statistically different among the groups (Figures 4(c) and 4(e)).

To elucidate the role of Fundc1-mediated mitophagy in HA pretreatment mitochondrial function, H9C2 cells were transfected with Fundc1-shRNA to knock down Fundc1 expression. qPCR revealed significant reduction in Fundc1 mRNA expression in transfected H9C2 cells (Figure 4(f)). Western blot analysis of H9C2 cells subjected to mimicking HA and/or OGD revealed that the Fundc1 and LC3-II expression in the OGD group was lower than that in the control and mHA+OGD groups (Figures 4(g) and 4(h)). The above findings were similar to those obtained in our animal experiments, in which MI suppressed Fundc1 expression and Fundc1-mediated mitophagy, but mHA treatment abrogated this effect. Further, the expression of Fundc1 in the Fundc1-shRNA+mHA+OGD group was lower than that in the control-shRNA+mHA+OGD group (Figures 4(g) and 4(h)). Simultaneously, knockdown of Fundc1 expression resulted in reduced LC3-II levels (Figures 4(i) and 4(j)). These results show that mHA pretreatment partially stabilized the expression of Fundc1 and promoted Fundc1-mediated mitophagy. Finally, no statistically significant changes in the expression of Pink1 and Parkin proteins were observed after the various treatments (Figures 4(i) and 4(k)). These data indicate that knockdown of Fundc1 expression destabilized the Fundc1 expression in the mHA group and downregulated LC3-II levels; however, this destabilization did not activate the compensatory mitophagy signaling via Pink1 and Parkin.

### 3.5. The Effects of Fundc1 Expression Changes in mHA on Mitochondrial Function, Cell Damage, and Apoptosis

**Figure 5:**
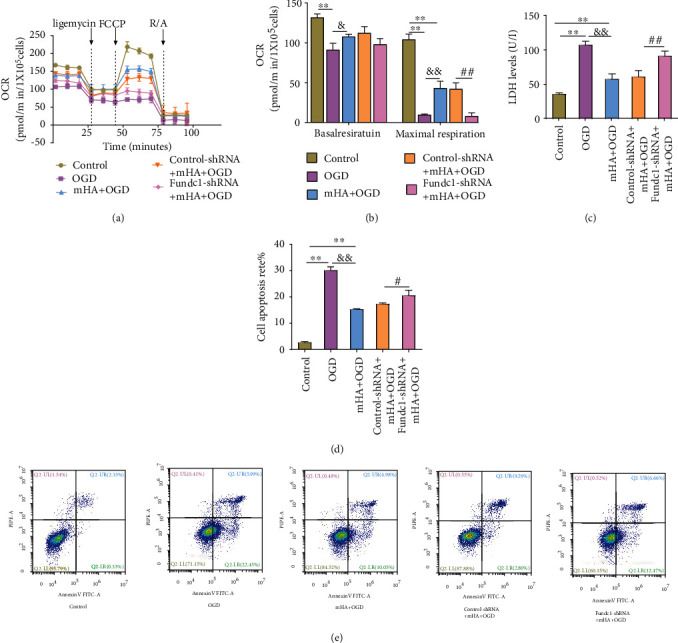
Mimicked hypoxia acclimation- (mHA-) mediated mitochondrial protection was abolished after knockdown of Fundc1 expression. (a, b) Measurement of mitochondrial oxygen consumption rate (OCR) in H9C2 cells using a Seahorse extracellular flux analyzer. Functional parameters of oxidative phosphorylation (basal respiration and maximal respiratory capacity) were calculated in the presence of mitochondrial inhibitors (*n* = 3 per group). (c) Lactate dehydrogenase (LDH) release by H9C2 cells after exposure to hypoxia (*n* = 4 per group). (d, e) Apoptosis rate, as determined by Annexin-FITC/PI staining (*n* = 3 per group). Data are expressed as mean ± SD. ^∗^*P* < 0.05, ^∗∗^*P* < 0.01 vs. control, ^&^*P* < 0.05, ^&&^*P* < 0.01 vs. OGD, ^#^*P* < 0.05, ^##^*P* < 0.01 vs. control-shRNA+mHA+OGD. FITC: fluorescein isothiocyanate; LDH: lactate dehydrogenase; mHA: mimicked hypoxia acclimation; OGD: oxygen glucose deprivation; OCR: oxygen consumption rate; PI: propidium iodide.

The basal oxygen consumption and maximal respiratory capacity of the mitochondria in cultured H9C2 cardiomyocytes in the OGD and mHA+OGD groups were significantly lower than those in the control group. Fundc1 knockdown attenuated the mHA pretreatment changes in the maximal respiratory capacity (Figures 5(a) and 5(b)). Finally, the LDH release and apoptosis rates in cardiomyocytes in the OGD group were significantly higher than those in both control and mHA+OGD groups. However, compared with the OGD group, the LDH release and apoptosis rates in cardiomyocytes in the mHA+OGD group were significantly reduced (Figures 5(c) and 5(d)). Fundc1 deficiency also significantly inhibited the antiapoptotic effect of mHA and increased the apoptosis rate and LDH release of H9C2 cells compared with those of the control-shRNA+mHA+OGD group (Figures 5(c)–5(e)). Collectively, these data suggest that Fundc1-mediated mitophagy can be activated by mHA and plays an important role in protecting cardiomyocytes from ischemia-hypoxic injury.

## 4. Discussion

Receptor-mediated mitophagy is a highly conserved cellular mechanism that selectively eliminates unwanted or dysfunctional mitochondria. It plays an important role in controlling mitochondrial quality in response to cellular energy needs and other mitochondrial and cellular cues [35]. Fundc1 contains three transmembrane domains, and its N- and C-terminal regions are exposed to the cytoplasm and intermembrane space, respectively [36]. Under normal conditions, Fundc1 can interact with the mitochondrial fusion protein OPA1 in the membrane space and is phosphorylated by CK2 at S13 and inactivated by SRC kinase at S18. During hypoxia, dephosphorylation of Fundc1 may be promoted by inactivating SRC kinase and CK2 and activating ULK1 and PGAM5 among other mechanisms. Dephosphorylated Fundc1 has a significantly increased affinity to LC3 and induces mitochondrial autophagy [16, 36–38].

Our results revealed that the expression of the Fundc1 protein and Fundc1-mediated mitophagy decreased under HF post-MI; meanwhile, mitochondrial function and structure are impaired, the myocardial contractility weakened, fibrosis was increased, and LV remodeling was impaired. These findings are consistent with those of a previous report showing that the lack of the mitochondrial receptor Fundc1 significantly aggravated myocardial remodeling, abnormal contraction, apoptosis, and other forms of cell death in HF [39]. However, HA treatment increased the expression of the Fundc1 protein in the LV myocardium, thus improving cardiac function after MI, reducing post-MI fibrosis, and delaying HF. These results indicated that MI inhibited Fundc1 expression and Fundc1-mediated mitophagy, while MI in combination with hypoxia acclimatization upregulated them. Wherefore, the difference in Fundc1 expression and the rate of Fundc1-mediated mitophagy between the MI and HA+MI groups may provide a clue about the mechanism through which HA improves cardiac function after MI. Therefore, to confirm the role of Fundc1 and Fundc1-mediated mitophagy, we knocked down the expression of Fundc1 in H9C2 cardiomyocytes and found that this knockdown abolished the protective effect of mHA on cardiomyocytes and increased the LDH release and apoptosis rates of cardiomyocytes. Previous studies that silenced Fundc1 expression in cells observed that the hypoxia treatment failed to induce mitophagy and that mitophagy could be reactivated by restoring the Fundc1 levels [40]. The above data suggested that HA may upregulate the expression of Fundc1 and Fundc1-mediated mitophagy in response to myocardial ischemic injury in mice, thereby increasing the tolerance of the heart to MI injury.

Recent studies have shown mitochondria to be dynamic organelles. When the mitochondrial network is damaged, mitochondrial quality control components are activated. Mitophagy, mitochondrial dynamics (biogenesis, fission, and fusion), and posttranslational modifications of mitochondrial proteins collectively control the mitochondrial quality [41]. Damaged mitochondria undergo fusion-fission kinetics to separate the unstable mitochondria with low membrane potential. Mitophagy degrades and eliminates abnormal mitochondrial components, while mitochondria capable of generating energy are further fused and repaired to maintain membrane potential and ensure the energy supply [5, 42, 43].

Mitochondrial oxidative phosphorylation reactions occur through the electron transmission chain, comprising four enzymatic complexes—I, II, III, and IV—in the inner mitochondrial membrane. Changes in the activity of the respiratory chain complexes, caused by various factors, directly affect cell homeostasis [44]. To better understand the effect of mitochondrial energy metabolism, we examined mitochondrial OCR using a XF 24 analyzer, which uses modulators of respiration that target components of the electron transport chain to reveal key parameters of the metabolic function. In the present study, we detected reduced mitochondrial RCR and maximal respiration in both the MI and HA+MI groups compared with those in the CON group, but the RCR and maximal respiration were higher in the HA+MI group than in the MI group, indicating that HA restored mitochondrial function impaired after MI [45]. Similarly, mHA treatment increased the mitochondrial basal and maximal respiratory rates in cardiomyocytes following myocardial ischemic injury. However, when Fundc1 was knocked down, the hypoxic preconditioning did not improve the mitochondrial function under ischemic damage, suggesting that Fundc1 played an important role in maintaining the mitochondrial function during myocardial ischemic injury. Furthermore, mitochondrial morphology is intimately linked to its function. The shape of a mitochondrion varies significantly depending on its metabolic status [46]. In the present study, electron microscopy revealed that the number of mitochondria decreased in the myocardial tissue of the MI group, but the area increased abnormally, and these effects were reversed in the HA+MI group, suggesting restoration of mitophagy and promotion of mitochondrial remodeling.

Recently, several studies have reported that Fundc1 may be involved in pathways related to mitochondrial fission and mitophagy in mammalian cells, namely, those mediated by CK2*α*, PGAM5, ULK1, and MARCH5 [37, 38, 47, 48], in response to hypoxia. Under hypoxic conditions, OPA1, CK2, and SRC kinases are separated from Fundc1, and PGAM5- and ULK1-mediated dephosphorylation enhances this effect. In addition, hypoxic stress can enhance the interaction between MARCH5 and Fundc1, leading to the ubiquitination of Fundc1 at K119 followed by its degradation, and desensitize mitochondria to hypoxia-induced mitophagy. Finally, Fundc1 binds to LC3 on phagocytes, helping them to phagocytose defective mitochondria via autophagosomes. These autophagosomes fuse with lysosomes to degrade and recycle mitochondria. In this study, we observed in animal experiments that HA treatment associated with stimulation of FUNDC1-mediated mitophagy was protective against myocardial ischemia-induced HF, while FUNDC1 knockdown partially reversed the protective effect of HA on MI cytotoxicity. However, whether the protective effect of HA treatment on MI-induced heart failure was reversed after FUNDC1 knockdown was not further confirmed in an in vivo model. Furthermore, specific signaling pathways upstream of Fundc1 under HA were not further explored, and autophagy agonists/inhibitors were not used in vivo or in vitro to respond to changes in FUNDC1 expression. Therefore, whether a specific pathway of Fundc1-mediated mitophagy under HA can reverse ventricular remodeling and cardiac function after ischemic injury requires to be elucidated by future studies.

## 5. Conclusion

In conclusion, the Fundc1 gene plays a critical role in the adaptation to chronic ischemia, such as in MI-induced HF. Upregulation of Fundc1 expression following MI appears to offer protection against cell death by enhancing Fundc1-mediated mitophagy. However, other studies have reported that excessive or prolonged activation of mitophagy may be detrimental and lead to cardiomyocyte mitochondrial DNA (mtDNA) loss. Therefore, further studies are necessary to better understand the mechanisms and regulatory events related to mitophagy in the myocardium. Appropriate induction of mitophagy could be a feasible strategy to treat cardiovascular diseases, including HF.

## Data Availability

The raw data supporting the conclusions of this article will be made available by the authors, without undue reservation.
